# Development of a 4Pi national involvement standards-based questionnaire to evaluate patient and public involvement

**DOI:** 10.1186/s40900-026-00852-1

**Published:** 2026-02-19

**Authors:** Kathryn Watson, Richard Morton, Maria Milenova, Hema Chaplin, Erin Letbe-Holder, Fiona Hackett, Bernadette Khoshaba, Kia-Chong Chua, Manuela Russo

**Affiliations:** 1 Health Service and Population Research Department, Institute of Psychiatry, Psychology and Neuroscience, King’s College London, London, UK; 2 Improvement Service, South London and Maudsley NHS Foundation Trust, London, UK; 3 Biostatistics & Health Informatics, Institute of Psychiatry, Psychology & Neuroscience, King’s College London, London, UK

**Keywords:** Patient and public involvement (PPI), Evaluation, Questionnaire development, Cognitive interviewing (CI), Co-design, Questionnaire appraisal system (QAS), Participatory quality improvement

## Abstract

**Background:**

Evaluation of patient and public involvement (PPI) is crucial to ensuring it is conducted with quality. However, determining how best to evaluate the experience of those doing involvement activities remains challenging, particularly given the complex landscape of diverse terminology and wide range of methods. There is a need for robust and accessible tools, grounded in established standards, that reflect the perspectives of patients and public contributors involved in PPI activities. To address this, we developed a questionnaire (4Pi Questionnaire) based on the 4Pi National Involvement Standards, a project that developed a framework around good practice and measurement, monitoring and evaluation of involvement activities.

**Methods:**

The questionnaire was developed through an iterative process involving literature review, experts’ input and extensive consultation with PPI contributors. Initial items were generated deductively from the five domains of the 4Pi National Involvement Standards framework (i.e., Principle, Purpose, Presence, Process and Impact). Subsequent versions of the questionnaire were refined using a face validity workshop, a Questionnaire Appraisal System, and focus group-based cognitive interviewing (CI) with PPI contributors. Feedback focussed on clarity, relevance, accessibility and questionnaire structure. There was a final round of testing to assess accessibility and usability.

**Results:**

The 4Pi Questionnaire is a structured measure designed to capture experiences of involvement in diverse contexts across healthcare and research. The questionnaire consists of an introduction followed by 31 items grouped into two sections A and B. Section A (“Your experience of involvement”) includes 22 items relating to a single recent involvement activity and cover the five domains of the 4Pi National Involvement Standards framework. Respondents rate their agreement with 19 statements using a five-point Likert scale, and answer two open-ended questions. Section B (“About you”) collects demographic information. Completion time is approximately 15–30 min. Involvement of subject-matter experts and PPI contributors ensured clarity of language, relevance of each question, and usability of the questionnaire.

**Conclusions:**

Grounded in the 4Pi National Involvement Standards, the 4Pi Questionnaire provides an accessible and acceptable tool to measure the experiences and perceptions of patients and public contributors doing involvement activities. Systematically developed with and for people with lived experience, the 4Pi Questionnaire addresses a gap in the availability of standardised, meaningful measures and supports efforts to embed inclusive involvement practices across healthcare and research settings.

**Supplementary Information:**

The online version contains supplementary material available at 10.1186/s40900-026-00852-1.

## Abstract (plain language summary)

Involving patients, carers and members of the public in shaping health services and research is important, as it can make healthcare more effective, respectful, and meaningful. However, it is not always easy to know whether involvement has been done well and in a genuine way. To help with this, a team of researchers and patient and public involvement (PPI) contributors, identified through service users and carers, and public involvement networks, co-designed a new tool called the *4Pi Questionnaire*. They were involved in defining the questions, their focus, language and number. The questionnaire is a short set of questions that can be filled out after taking part in an involvement activity, for example, helping to plan services, joining steering groups, or being on interview panels. The questionnaire asks about people’s experiences and their views, such as whether they felt respected, understood their role, had enough support, and felt their input made a difference. The questionnaire is based on a framework called the *4Pi National Involvement Standards*, which set out what best practices for PPI should look like. The 4Pi Questionnaire is intended to help organisations learn how to improve involvement and give people the chance to have their voices heard in a meaningful way.

**Supplementary Information:**

The online version contains supplementary material available at 10.1186/s40900-026-00852-1.

## Background

Patient and public involvement (PPI) in research refers to an activity done ‘with’ or ‘by’ patients and the public rather than ‘about’, ‘on’ or ‘for’ them [1]. It involves the active participation of patients, carers, and members of the public as partners throughout the research process, rather than as research participants. There are compelling ethical and practical reasons for embedding PPI into research: people have the right to shape studies that concern their lives and health; their lived experiences help make research more relevant and impactful; and involvement fosters greater accountability and transparency of research [2]. In the UK, policies and legislation promoting PPI in health and social care have been in place since 1980s [3]. Within care and service-improvement contexts, alternative terms to PPI are used such as ‘service users (SUs) and carers’ or ‘people with lived experience’ which emphasise individuals’ roles as recipients and partners in health and social care services [4]. As this study was developed in a research context, PPI is used as an overarching descriptor, and reflects the methodological framing of this work and its alignment with established research standards and reporting expectations. Where relevant, specific roles (e.g., ‘contributors’) are clarified.

Over time, national funding bodies and healthcare organisations have moved from encouragement to expectation, now routinely mandating PPI in research, care planning and service improvement (NIHR UK Standards for Public Involvement 2019). Such developments have led to a rapid rise of PPI in the UK and internationally [5]. Yet, meaningful PPI remains difficult to achieve in practice as the involvement of people with lived experience is often experienced as tokenistic, with limited influence on decision-making and insufficient recognition of contributors’ perspectives [6]. As such, improving practice and learning from what works and why is crucial [7, 8].

Over the past two decades, efforts to evaluate PPI have increased [5, 9, 10]. Reviews of this work have revealed a complex and fragmented landscape, with a wide range of terms, methods, and tools, many of which are seldom used beyond the groups that develop them. A lack of scientific rigour, unclear focus, issues with accessibility, relevance, concerns over duplication of effort, and misalignment with key dimensions of engagement may contribute to this [5, 9]. Patients and public members are also rarely involved in the development of such evaluation tools [9] and uncertainty remains about which aspects of PPI should be prioritised for evaluation [10]. Taken together, these challenges highlight the need for more robust, usable, and inclusive approaches to evaluating PPI activities by gathering information from those who are directly involved.

We identified the 4Pi National Involvement Standards, often referred to as the “4Pi framework” [3], as a valuable resource. The 4Pi framework provides a set of national standards for good practice and high-quality involvement, with relevance across research, service delivery and policy. Although developed by and with mental health service users and carers, the guiding principles are extendable to other populations and contexts, including research. The 4Pi framework covers five domains: Principles, Purpose, Presence, Process and Impact – and emphasises shared values, inclusive participation, and meaningful collaboration (Table 1). Recognising its clarity, acceptability, and grounding in lived experience, we selected the 4Pi framework as the basis for developing a new tool to evaluate involvement from the perspectives of individuals directly involved in the activity.

**Table 1:** Description of the 4Pi National involvement standards domains by Faulkner et al. 2014 [3]

4Pi Domain	Description
Principles	Meaningful and inclusive involvement relies on a commitment to shared principles and values, such as respect, inclusivity, equality, fairness, and recognition of the knowledge that people with lived experience bring.
Purpose	Involvement should have a purpose that is clear and meaningful to everyone involved. These should be agreed and communicated at the beginning.
Presence	Involvement should take a diverse and inclusive approach and should be present at all levels and all stages of an organisation and within projects, including at decision-making levels. It addresses whether the right people are included and represented, and whether barriers to involvement are mitigated or removed to facilitate contribution from everyone involved.
Process	Planning is essential for successful involvement, and should cover recruitment and engagement, communications, appropriate support, training and payment. It addresses how involvement is conducted in practice.
Impact	Involvement should ultimately bring an improvement to people’s lives. Impact should be explored at different levels, including ethos and culture, policy and planning, delivery of the project, outcomes, diversity and equality opportunities, and experience of services. This also consider whether feedback is provided after completing the involvement activity so that people involved can see how their input has been used.

In this article, we introduce a new questionnaire, referred to as the 4Pi Involvement Experience Questionnaire or simply 4Pi Questionnaire, which aims to assess service users’ (SUs) and carers’ views on their experiences of undertaking PPI activities across the five domains identified by the 4Pi framework (Table 1). Specifically, it measures perceived fairness, clarity, support, influence and contribution – core components of meaningful involvement as defined by the 4Pi framework. Although the 4Pi framework was originally developed in the field of mental health, the domains are not only applicable to mental health. In the same way, the 4Pi Questionnaire can be used to measure experience across a wide variety of contexts and different levels of involvement. Its potential applications range from healthcare improvement activities, to research, policy development, and education.

The aim of this work is therefore: (1) to offer a robust, relevant and meaningful tool, which can be used to measure experience of PPI contributors (or SUs and carers depending on the context wherein the questionnaire is used) to support the evaluation of PPI activities; (2) to describe the different steps underpinning the co-design of the questionnaire and of the guidance and recommendations for its use. PPI contributors were involved in the design of the questionnaire to ensure that their lived experience shaped it. This improved the questionnaire’s relevance, accessibility, and subsequently will enhance its future impact.

## Methods

### Study design

The 4Pi Questionnaire was co-designed by a team of researchers, PPI co-ordinators, PPI contributors, and the Service User and Involvement Lead at South London and Maudsley (SLaM) National Health Service (NHS) Foundation Trust. The team adopted a ‘what will work best’ approach to produce a self-report questionnaire that was accessible, appropriate, meaningful and feasible for application to a wide variety of contexts.

Following domain identification and item generation, iterative cycles of questionnaire review and modification were carried out (Table 2). As described by relevant literature [11, 12], the following steps were followed to develop the questionnaire: objective setting, gathering input from experts and target population focus groups to develop key concepts, drafting survey questions, checking the fit between questions and objectives, expert appraisal, focus group- based cognitive interviewing (CI), and producing a field test version of the questionnaire.

**Table 2:** Questionnaire development steps

Step	Name of development step	Description
1	Item development: framework mapping and item generation	Item development combined: (a) review of literature (MR), relevant to existing evidence about measuring experience of doing involvement activities; and (b) direct experience (RM) of needs emerged in the contexts of leading service users and carers involvement activities at a National Health Service (NHS) Trust.
2	Subject matter expert consultation: initial review	Consulting (MR, HC) with a subject-matter expert (*n* = 1) to assess alignment of draft items with the underlying framework domains.
3	PPI consultation: face validity	An online consultation (MR, RM, HC, ELH) with PPI contributors (*n* = 4) to explore if initial draft of items were measuring what they were supposed to measure.
4	Questionnaire Appraisal System (QAS) review	An evaluation (KW), using an adapted version of the “modified QAS” (Schaad 2020), referred to as adapted QAS, for clarity and structure. This helped to find and fix common questionnaire problems.
5	Subject matter expert consultation: item refinement	Consulting (KW) with subject-matter experts (*n* = 2) to gather opinions about the refined list of items to be discussed in the focus group-based cognitive interviewing (see Step 6).
6	Focus group-based Cognitive Interviewing (CI)	Online focus group (KW, MR, ELH) to gather opinions from PPI contributors (*n* = 5) about the appropriateness of items along with overall questionnaire clarity.
7	PPI consultation: accessibility and usability	Consulting (KW, MR) with PPI contributors (*n* = 11) to identify if final draft questionnaire met expected respondents’ needs in terms of accessibility and usability.

### Item development: framework mapping and generation (step 1)

This step involved identifying key contents and generating candidate items aligned with the five 4Pi framework domains. The identification of relevant items to be included in the questionnaire combined a deductive approach which involved review of relevant literature to explore key issues about conducting meaningful involvement, and an inductive approach which relied on knowledge and input from subject matter experts and individuals with lived experience (i.e., PPI contributors) with direct experience of doing involvement activities in research and healthcare context.

### Subject matter expert consultations: initial review (step 2) and item refinement (step 5)

Three subject-matter experts were identified across two steps. They were chosen based on their reputation, and knowledge in the field. These included one co-author of the 4Pi framework (Step 2) to guide high level mapping of potential items to domains, and two PPI leads at the collaborating NHS Trust who provided more focused feedback on the individual items in an advanced draft version of the questionnaire (Step 5; Table 2).

### Questionnaire appraisal system (QAS) review (step 4)

Тhe questionnaire review was conducted using the Questionnaire Appraisal System (QAS) approach, which is a checklist designed to aid finding and fixing common questionnaire problems [11]. The “modified QAS”, developed by Schaad et al. 2020 [13], covering common problematic question characteristics [14] with issues arising in cross-cultural and translated surveys [15], , reviews both flow of the questions and individual questions. For application to our self-report questionnaire, an adapted version was created by the study team and referred to as “adapted-QAS”.

### Focus group-based cognitive interviewing (CI) (step 6)

Cognitive interviewing (CI) is a qualitative method used in questionnaire development to evaluate how respondents understand, interpret, and respond to survey questions [16]. Although CI is usually conducted individually, focus group-based CI was shown to be a valid method for questionnaire cognitive pre-testing, and can more effectively promote open dialogue among participants and produce richer data, compared to traditional one-to-one CI [17, 18]. The objective of the focus group-based CI was reparative, that is a “find and fix” approach to identifying major sources of survey response errors [11, 16]. This information was then used to guide the 4Pi Questionnaire modification.

Purposive sampling, through the involvement registry (listing individuals who completed involvement activities) at the collaboration NHS Trust, was used to identify the participants. Invitations were sent via email by the research team. Eligibility criteria included being at least 18-years-old and having experience of involvement at the NHS Trust. Also, participants needed to be naïve to the questionnaire under development. To ensure diversity and variety of opinions demographic characteristics were considered where composing the sample. To enhance accessibility and inclusivity, the focus group-based CI activity was carried out online and participants were posted or emailed ahead of the workshop relevant materials according to individual preferences. Informed consent was obtained prior to the focus group-based CI. Participants were paid in line with National Institute for Health and Care Research (NIHR) guidelines [19].

Procedure: At the beginning of the focus group-based CI, participants were asked to complete the 4Pi Questionnaire independently. Instructions were given to record start and finish times, note any comments, item problems and revision suggestions. A researcher from the team (KW) led retrospective cognitive probing with the group. This involved exploring participants’ views and experience of the questionnaire after they completed it. The questionnaire was reviewed in a sequential manner, and within each section participants were first asked about issues that were pre-identified by the research team as requiring further exploration. Following this, participants were given time to raise any additional issues. However, those issues that were felt to be a lower priority for response error risks were not probed directly due to time constraints. As such, not all questions were probed. The total duration of the focus group-based CI was three hours.

As mentioned above, the cognitive probing followed a semi-structured approach, consisting of pre-planned (proactive) and spontaneous (reactive) verbal probes (Supplementary material A). The adapted-QAS was used as a drafting framework for preplanned (anticipated and optional) probes. Probe guidance [11] was followed, for example ensuring probes are balanced, avoiding speculative probes and being conservative about the number of probes asked. Probes were designed to explore the meaning of specific terms and phrases to participants, how well questions were understood and answered, what information the question is capturing in practice, if response categories were adequate. Spontaneous (reactive) probes were initiated in the focus group-based CI where required.

Participants were given up to a week after the CI workshop to add any additional thoughts to their notes, after which they were then returned to the research team for analysis. Completed questionnaires were not collected because that was not the purpose of the CI workshop. Two co-facilitators (MR, ELH) took notes during the CI workshop which was also audio-recorded, so that targeted, anonymised verbatim transcriptions could be made available to supplement notetaking if required.

Informal data analysis [11] was conducted (KW) in Microsoft Excel. Anonymised participant data was summarised at the group level and interpreted in light of feedback and modifications from earlier development steps. Validation of data extraction and analysis was achieved through review and discussion within the research team.

### PPI consultations: face validity (step 3) and accessibility and usability (step 7)

Face validity: Four PPI contributors were identified through network mapping supported by one the co-authors and Lead for Service User and Carer Involvement at SLaM (RM) NHS Foundation Trust, via the involvement registry who lists individuals who completed involvement activities in the Trust. They participated in the face validity (the degree to which the questionnaire is measuring what it is intended to measure) via online group consultation. This group was also involved in a final review of an accompanying guidance to implement the 4Pi Questionnaire and in dissemination activities.

Accessibility and usability: Twelve potential PPI contributors, identified through PPI network mapping and naïve to the final draft questionnaire, were invited via email by RM to participate in a small testing aiming at ensuring accessibility and usability of the questionnaire (Step7). Eleven of them accepted the invite. They completed the questionnaire (no data was collected) and provided feedback on it via an online or postal form. To increase accessibility during this consultation, one individual was enabled to provide feedback via a telephone conversation.

### PPI contribution to the study

An internal and external group of PPI contributors were involved in the study. The internal group (six members of the research group’s general PPI group) were updated about progress over the course of the study via monthly meetings. The external group (four individuals who had been involved in PPI activities at the collaborating NHS Trust and with the research group) were involved in development of the 4Pi Questionnaire (Step 3; Table 2) and its dissemination. They were identified via network mapping and needed to have experience of involvement at the collaborating NHS Trust or with the research group. The GRIPP2-Short Form checklist for reporting of PPI in health and social care research is provided as Supplementary material (C).

## Results

The 4Pi Questionnaire is the main outcome of this work. It consists of an introduction and 31 items subdivided into two sections, A and B (Supplementary material B). Specifically, the introduction contains background information to help respondents decide whether they wish to complete the questionnaire. Section A, ‘Your experience of involvement’ is made up of 22 items: 19 statements for which respondents rate their level of agreement, and two, non-mandatory, open-ended questions. The items are designed to capture experiences related to a *single* recent involvement activity whose type they are asked to specify in Item 1.

The 19 statements for which respondents rate their level of agreement is carried out using a five-point Likert scale from ‘strongly disagree’ [1] to ‘strongly agree’ [5]. These statements relate to the 4Pi domains as follows: items 2–4 map to Principles, 6–8 to Purpose, 9–10 to Presence, 11–17 to Process, and 18–20 to Impact. All items are required to be completed; however, the responses ‘prefer not to answer’ and ‘not applicable’ are options for all items. Also, as the terms ‘SUs and carers’ may be preferred by future respondents, these are used instead of ‘patients and public members’ within the questionnaire. The two open-ended questions offer the possibility to share additional information about the experience of involvement and aspects which the respondent felt that were not covered by the multiple-choice questions. Section B, ‘About you’ (9 items), aims at collecting demographic information. Completion time is estimated to be approximately 15–30 minutes.

Several versions of the questionnaire were created before determining the final version presented in this work. This followed an intensive iterative process wherein suggestions and feedback were discussed within the team, consulted with subject matter experts and experts by lived experience, before embedding into a new version of the questionnaire. For instance, while the initial versions had between five and six statements mapping onto each domain, so totalling above 30 statements, the final version includes 22 statements. A detailed description of how each of the steps described in the Methods section contributed to the development of the questionnaire is reported below.

### Steps 1–2: item development and expert review

As stated in the Introduction, the development of the questionnaire was guided by the existing 4Pi framework. Therefore, our domains were pre-defined. The definition of each domain (i.e., Principle, Purpose, Presence, Process and Impact), which was based on published documents [3] and the direct experience of conducting PPI activities, guided the development of the first draft questionnaire. Next, consultation with an expert on the 4Pi framework informed the identification of salient concepts to be covered by the questionnaire statements.

### Step 3: face validity

As a result of the online face validity (group) consultation with four PPI contributors, the draft questionnaire was refined. Feedback primarily focused on improving the clarity of statements and their intended meaning, thereby ensuring relevance and alignment with the corresponding 4Pi domain. Also, the group agreed in saying that overall there were too many statements which could burden respondents, therefore they suggested to keep statements to a reasonable number.

### Steps 4–5: pre-cognitive interview results

The main issues that were identified during these phases related to the overall questionnaire format and structure (such as, issues with layout, item grouping, number of items) and individual items that were checked against the adapted-QAS (i.e. issues with encoding, clarity, assumptions, sensitivity, and response categories). Key modifications included: section merging and reordering; changes to the introduction, domain headings and explanatory text; item rephrasing, reordering and reduction; adding missing items and response categories. Items were removed according to the following criteria: significant overlap with other items; respondents unlikely to have the knowledge to answer the item; items more suited to other methods of data collection; low priority weighed against respondent burden; domain irrelevance.

### Step 6: cognitive interviewing results

Five participants, out of nine recruited, took part in an online focus group-based CI. Participants were all females, representing a mix of ages (*n* = 1 between 31 and 40 years old; *n* = 1 between 51 and 60; *n* = 3 between 61 and 70) and ethnic backgrounds (*n* = 3 from Black background; *n* = 2 from White background; *n* = 1 from Asian background). All participants completed and/or were currently doing some involvement activities at the time of the workshop.

The main findings and modifications included issues with the scope, accessibility, response categories and questionnaire flow that were addressed in each part of the questionnaire such as Introduction, Sections A and B (Table 3). Most participant suggestions were actioned. Reasons for not actioning some suggestions included the following: issue addressed already, irrelevant following other modifications, not aligning with best practice, or too great an impact on questionnaire length.

**Table 3:** Focus group-based cognitive interview results

Location in the questionnaire	Category	Code	Participant comments	Modifications
Introduction	Accessibility	Completion times	Give a range of completion times so people don’t feel “inadequate” if they take longer than the stated time	Updated timings to approximately 15 to 30 min, to align with completion timings gathered as part of CI
		Support	Some people might need more support to complete the questionnaire	Updated introduction to include signposting for additional support
Section A: Involvement experience	Scope	Focus	Difficulty expressed and different methods used to generalise across more than one activity.	Focus changed to asking respondents to focus on a single activity.
	Accessibility	Instructions	Adding domain descriptions would be helpful. Of note, earlier PPI feedback on questionnaire version 2 stated that short domain descriptions were too “heavy” and potentially confusing.	Comment redundant as domain groupings and associated text removed to reduce length of questionnaire.
		Clarity	Vague or confusing item wording	Items rephrased for clarity e.g. replaced *transparent* with *open*, replaced *project* with *activity*, changed *I had information in a format that met my needs* to *I had information that was easy to read and access.* Examples added to some items e.g. For item *I had enough support to manage how I felt*, the following examples were added: *help with preparing for meetings*,* help with how to share my story*,* having time and space after sharing my story to reflect and talk*,* being able to leave and come back to a meeting if needed.*
		Assumptions	Split double-barrelled items	Not actioned, as splitting double-barrelled items would significantly lengthen questionnaire and participant feedback suggested items were nevertheless understood.
	Response categories	Likert scale	Likert scales may be easier to answer if using levels of frequency instead of levels of agreement when generalising across more than one activity.	Levels of agreement more appropriate when asking respondents to focus on a single experience. Therefore, Likert scales kept to levels of agreement.
	Other	Item relevance	Items not always specific to the 4Pi framework	Items reviewed to confirm relevance to 4Pi domains
Section B: About you	Flow	Item order	Ask *About you* questions at the beginning of the questionnaire. Of note, following earlier PPI feedback on questionnaire version 2, *About you* questions were moved to the end of the questionnaire.	Items about indicating the type of involvement activity moved to the beginning of Section A. Other *About you* questions kept to Section B.
	Response categories	Clarity	Confusing, inaccurate or missing.	Confusing and inaccurate response categories removed. Missing response category added.
Overall	Scope	Add additional items	Suggestions to include additional items for investigation e.g. identifying the proportion of projects where involvement has taken place, identifying whether SUs and carers experienced any barriers to becoming involved, identifying at what level and to what extent SUs and carers were involved throughout projects, how long individuals have been doing involvement.	Suggestions not included in questionnaire as outside of current scope and would further add to questionnaire length. Suggestions included in guidance accompanying final questionnaire as examples of areas that could be investigated as part of a wider evaluation or follow-up evaluation. One suggestion about how long individuals have been doing involvement was not included in the guidance, as this could potentially create prejudice in data interpretation.
	Accessibility	Length	Questionnaire too long.	Reduced length of questionnaire by shortening the introduction and removing domain groupings and associated text. Reduced number of items by removing domain-specific free-text boxes.
		Font	Use larger font size	Font changed to Arial size 12, to align with accessibility standards (see https://www.gov.uk/guidance/publishing-accessible-documents)
		Clarity	Use plain English	Introduction and all items reviewed and edited where relevant, to further simplify language e.g. *I had enough emotional support* rephrased to *I had enough support to manage how I felt*.
		Improvements	Suggestions to improve accessibility during questionnaire administration e.g., include a progress bar on online versions, include suggestions to providers in providing different ways respondents could be supported to provide feedback, such as a one-to-one conversation going through the questionnaire	Suggestions included in guidance accompanying final questionnaire. Questionnaire reviewed and edited to comply with accessibility standards (see https://www.gov.uk/guidance/publishing-accessible-documents) Accessibility checked using Accessibility Checker in Microsoft Word and all issues addressed.
	Response categories	Check boxes	Add check boxes on electronic word documents	Check boxes added

One of the most relevant issues was around the possibility of allowing an average rating to reflect more than one experience. Although limiting responses to a single activity might represent a burden for individuals completing more than one activity, this decision was made to facilitate the rating process and to help overcome not only the challenge of recall bias, but, more importantly, the difficulty of identifying a single rating that fits different experiences, thereby avoiding neutral responses (e.g. between one positive and one negative experience). Table 3. Focus group-based cognitive interview results.

### Step 7: accessibility and usability review results

All PPI contributors felt the length of the questionnaire was “about right” and that it was “easy” or “very easy” to understand and answer. On their experiences of completing the questionnaire, one individual said: “*The questions themselves were excellent and I understood everything clearly*”, while another said, “*Overall*,* I think it is well thought out and very fair in the type of questions that it asks. However I think there is merit in maybe taking a broader approach*,* than just asking someone about one experience*.”

The main areas identified for improvement related to the challenge of limiting responses to only one involvement activity, clarity, response categories, accessibility and scope of questionnaire. Key modifications included changes to the introduction (adding clarity), incorporating examples to several items, item rephrasing, rephrasing and adding missing response categories, including additional information in accompanying questionnaire guide.

The most significant modifications included reducing the questionnaire length by removing domain headings and groupings, and removing a free-text box at the end of each domain. Two free-text boxes were retained at the end of Section A. In addition, the focus of the questionnaire was changed from asking respondents to generalise across more than one activity, to answer based on their experiences of a single involvement activity.

A Questionnaire Guide was also produced to accompany the 4Pi Questionnaire (Supplementary material D). The Guide consists of an introductory section about the background of the work, followed by a description of the questionnaire development, and the guidance and recommendations for administering the questionnaire.

## Discussion

This study presented the 4Pi Questionnaire which is an accessible and acceptable evaluation tool, developed through a systematic and rigorous process, aiming at measuring the experience of PPI contributors involved in the planning, delivery and evaluation of healthcare services and research.

The 4Pi Questionnaire represents an advancement to available methods to evaluate involvement activities as the field was felt to be lacking particularly in terms of standardisation, scientific rigour, and accessibility [9]. As such, the 4Pi Questionnaire is well-placed as a PPI experience-based evaluation tool, across a wide variety of contexts. However, the 4Pi Questionnaire is not intended to capture the wide range of contextual, relational, and organisational factors which shape a successful involvement. Rather, it is designed to capture individual-level experiences of involvement, focusing on how respondents perceive key dimensions of involvement within a specific activity. Therefore, instead of being a standalone measure of involvement quality, this tool could be used to complement broader (mixed-methods) evaluation approaches.

The 4Pi framework is a versatile set of standards [20–22] that has also been suggested as a valuable approach to guide PPI evaluative efforts [3, 6, 21]. As far as we are aware, the 4Pi Questionnaire is the first example of a PPI evaluation questionnaire that has been developed using the 4Pi framework. It is worth noting the differences between the 4Pi Questionnaire and other measures aiming to assess experiences of stakeholders or public involvement, such as the Goodman et al.’s Qualitative Community Engagement (QCE) measure [23]. The QCE measure is designed primarily for use by research teams to quantify levels of community engagement in research projects. It focuses on structural elements of engagement (such as, community influence on study design, resource allocation, shared decision making) and process elements like quality of communication, responsiveness to community priorities. It is intended to help track and compare how engagement is implemented across different studies. The 4Pi Questionnaire in contrast, was developed to capture the personal experiences of individuals involved in healthcare service improvement or research activities. It draws from the 4Pi National Involvement Standards and focusses on lived experience principles such as feeling respected, understanding one’s role, and receiving support. Rather than assessing engagement from an organisational or project’s lens, it explores whether involvement felt meaningful, inclusive, and impactful from the participant’s perspective.

The 4Pi Questionnaire was co-designed with PPI contributors from the start of the project (i.e., items’ identification) to its end (i.e., testing accessibility and usability), thus putting a strong emphasis on accessibility, emotional support, and inclusive participation. These features are less prominent in the QCE measure which, as mentioned above, primarily assesses engagement at a more structural and system level. Therefore, the two instruments reflect distinct (but complementary) approaches to evaluate engagement (i.e., at individual, experiential level the 4Pi Questionnaire, and at community level the QCE).

During the questionnaire development process, the main issues identified aligned with general CI outcomes reported elsewhere [11]. One of the main challenges we encountered was how best to deal with the inherent variation of involvement activities. For example, these can range in type, from one-off to long-term, individual to group, and consultation to co-production. Respondents may also vary in the number of activities they choose to get involved in, as well as in the extent of their involvement. The decision to focus on a single involvement activity, rather than to ask respondents to generalise their experiences across multiple activities, was made for two reasons. This approach will enable experiences to be stratified by activity type. It was also done to ease questionnaire completion and improve the meaningfulness of responses. However, some individuals, particularly those involved in many involvement activities, may still find this approach challenging. As such, we recommend evaluators consider accepting multiple questionnaire submissions to enable respondents to feed back about more than one activity, if desired.

Another key challenge was achieving the right balance between comprehensiveness (satisfactory representation of the 4Pi framework and expectations of SUs and carers) and respondent burden (the amount of effort required by respondents to complete the questionnaire). Respondent burden mostly related to item complexity and questionnaire length. PPI contributors involved in the final round of questionnaire review suggested that the questionnaire was easy to understand and complete. They were also satisfied with the questionnaire content, which, like other PPI evaluation tools, covers elements related to perceived empowerment, impact, respect, support and value [5]. However respondent burden may still be an issue for some. Arguably, removing and simplifying further items may compromise the questionnaire’s ability to meaningfully represent the 4Pi framework and the key dimensions of involvement.

We believe the 4Pi Questionnaire would add most value when used regularly, and as part of a repertoire of approaches to provide a comprehensive evaluation of PPI. Complementary approaches include alternative methods of generating and collecting data, such as discussion-based methods, approaches to collect views from additional perspectives, including staff members, and tools that can measure observable impacts on research outcomes. We hope that the 4Pi Questionnaire as a standardised, accessible and acceptable tool for evaluating PPI activities will help to improve the frequency and quality of involvement activities’ evaluations. The information collected through the 4Pi Questionnaire can be used by service providers (e.g. the NHS, charities), organisations (e.g., research institutions, universities, Voluntary, Community, and Social Enterprise), or individuals who carry out involvement activities and wish to evaluate how these are conducted. It can be used as part of a quality improvement project, service evaluation, monitoring report, or research study, either as a standalone feedback tool to measure individual experience of those individuals involved in the activity or alongside other qualitative and quantitative evaluation methods.

### Strengths and limitations

This study has some limitations that need to be acknowledged. Due to time and resource constraints only one focus group-based CI was carried out with a small sample size of female participants. However, this limited sample was complemented by additional input from PPI contributors at other stages of the development process. Although PPI participation was carefully planned to ensure that everyone could contribute, feel valued, and be acknowledged for their work, due to limited capacity of the research team to provide preparatory training, PPI contributors were not able to be fully involved in the analysis of the focus group-based cognitive interviews. Completed questionnaires were not analysed, which limited analysis of response error. In addition, psychometric testing was not undertaken. To increase the validity and reliability of the 4Pi Questionnaire, as well as psychometric testing [12], additional development steps could be carried out, such as conducting a pilot test with a diverse cross-section of the target population [24]. In turn, the scoring of each domain should be taken with caution and not as an indicator of ‘good’ and/or ‘bad’ involvement. However, this can be used for providers to explore specific involvement aspects (i.e., the five domains of the 4Pi framework), thus identifying areas of strength and weakness in the way involvement is carried out to inform future quality improvement initiatives. The strengths of the 4Pi Questionnaire are attributable to its foundation in the 4Pi National Involvement Standards and its extensive development process, which involved both subject-matter experts and PPI contributors at various stages and levels. This development process has been transparently described to illustrate the scientific rigour employed. We also share guidelines and recommendations (i.e., Questionnaire Guide), which draw on feedback from PPI contributors and subject-matter experts, to improve the questionnaire’s accessibility to other evaluators and to facilitate its implementation. The focus group-based CI provided rich insight on the clarity, accessibility, and relevance of the tool. Key areas for improvement included amplifying language, clarifying item wording, and ensuring that questions reflecting a single involvement experience rather than multiple activities. Participants taking part in the focus group-based CI were from diverse backgrounds. Furthermore, these participants reported feeling satisfied with their ability to take part and their overall experience of the CI. Together, this supports success of the CI in capturing valuable insights and suggestions to improve the 4Pi Questionnaire development. Furthermore, the iterative approach to overall questionnaire development reflects reviewers’ views, enhancing the reliability and robustness of the design.

## Conclusions

The 4Pi Questionnaire is a 31-item evaluation tool that can provide a universally relevant, rigorous, accessible and acceptable means to measure SUs and carers experiences of involvement across a wide variety of contexts. The 4Pi Questionnaire involved both PPI contributors and subject-matter experts to enhance its accessibility. It could be a valuable tool as part of a broad (mixed-methods) evaluation, to complement other qualitative and contextual approaches, and support the evaluation of PPI involvement experience at any stage of a project, across different contexts and level of engagement.

## Supplementary Information

Below is the link to the electronic supplementary material.

Supplementary Material 1

## Data Availability

No datasets were generated or analysed during the current study.

## Abbreviations

CI: Cognitive interviewing
NHS: National health service
PPI: Patient and public involvement
QAS: Questionnaire appraisal system
SLaM: South London and maudsleySus: service users
