# Correction: Development of a 4Pi national involvement standards-based questionnaire to evaluate patient and public involvement

**DOI:** 10.1186/s40900-026-00936-y

**Published:** 2026-07-06

**Authors:** Kathryn Watson, Richard Morton, Maria Milenova, Hema Chaplin, Erin Letbe-Holder, Fiona Hackett, Bernadette Khoshaba, Kia-Chong Chua, Manuela Russo

**Affiliations:** 1 Health Service and Population Research Department, Institute of Psychiatry, Psychology and Neuroscience, King’s College London, London, UK; 2 Improvement Service, South London and Maudsley NHS Foundation Trust, London, UK; 3 Biostatistics & Health Informatics, Institute of Psychiatry, Psychology & Neuroscience, King’s College London, London, UK

**Correction to: Res Involv Engagem (2026) 12:48.**

10.1186/s40900-026-00852-1

Following publication of the original article [1], the authors identified an error in the name of Hema Chaplin. The given name was incorrectly indicated.

The incorrect author name is: H A. Chaplin.

The correct author name is: Hema Chaplin.

The author group has been updated above and the original article [1] has been corrected.
