# Supplementary material for: Development of a 4Pi national involvement standards-based questionnaire to evaluate patient and public involvement
Source: Res Involv Engagem. 2026 Feb 19;12:48. doi: 10.1186/s40900-026-00852-1 (PMC13097933; doi:10.1186/s40900-026-00852-1)
Supplement: Supplementary file 1 — Supplementary Material 1 [file 40900_2026_852_MOESM1_ESM.docx]

**Supplementary material**

1. **Verbal probing guide.** Verbal probing guide for 4Pi Questionnaire version 5 used during the focus group-based cognitive interview.

**Individual question probes**

| **Section/Domain** | **No.** | **Question** | **Probes** |
| --- | --- | --- | --- |
| A/ Principles | 1 | I felt respected and included. | What does the term "included" mean to you? |
|  | 3 | Service users and carers had the power to influence how things were done. | What were you thinking about when you answered this question?  [If required] What does the term "power" mean to you? |
|  | 4 | There was a transparent and honest approach to doing things. | What were you thinking about when you answered this question? |
|  | [End of *Principles* group] | | Any other issues related to the *Principles* group of questions that you would like to talk about before we move on to the next group? |
| A/ Purpose | 6 | I understood everyone’s roles, expectations and goals. | What were you thinking about when you answered this question? |
|  | 7 | I understood what I could and couldn’t influence. | What were you thinking about when you answered this question? |
|  | [End of *Purpose* group] | | Any other issues related to the *Purpose* group of questions that you would like to talk about before we move on to the next group? |
| A/ Presence | 10 | Service users and carers were involved throughout the overall project. | What does the phrase "overall project" mean to you? |
|  | 11 | Service users and carers had different backgrounds and experiences that were relevant to the activity. | Was this question easy or difficult to answer? |
|  | [End of *Presence* group] | | Any other issues related to the *Presence* group of questions that you would like to talk about before we move on to the next group? |
| A/ Process | 16 | I had enough practical support. | What you understand by the term "practical support"? |
|  | 17 | I had enough emotional support. | What you understand by the term "emotional support"? |
|  | 18 | I was rewarded appropriately. | What does the term "rewarded" mean to you? |
|  | [End of *Process* group] | | Any other issues related to the *Process* group of questions that you would like to talk about before we move on to the next group? |
| A/ Impact | 22 | My contribution made a difference to the activity. | What were you thinking about when you answered this question? |
|  | 23 | My contribution made a difference beyond the activity itself. | Was this question easy or difficult to answer?  [If required] What do you understand by the phrase “beyond the activity”? |
|  | [End of *Impact* group] | | Any other issues related to the *Impact* group of questions that you would like to talk about before we move on to the next section? |
| B | 27 | Which activities were you involved in over the last 3 to 6 months? | How easy or difficult was it to choose answers from the list?  [If required] Do you think these are the right types of activities to list for this question? |
|  | [End of *About you* section] | | Any other issues related to the *About you* group of questions that you would like to talk about before we move on to the last set of questions which refer to issues to do with the overall questionnaire? |

**Overall questionnaire probes**

| **Location of issue** | **Probe** |
| --- | --- |
| Section A | If you had mixed good and bad experiences, how did you approach completing the questionnaire?  [If required] For example, maybe your answers reflected one particular experience that felt important to you, what happened most of the time, or maybe you chose a mid-way point in-between both good and bad experiences?  [If required] If you had mixed good and bad experiences, how did you come up with your answer for Question 21. “I had a positive experience”? |
|  | Section A asks about your experiences of involvement over the last 3 to 6 months. Did this timeframe feel too short, about right or too long? |
| [End of O*verall questionnaire* questions] | Any other issues related to the *overall questionnaire* that you would like to talk about before we come to the end of the workshop?  [If time permits]  Did you find the questionnaire length too short, about right or too long?  Did you find the question grouping helpful or unhelpful?  Did you find the question group subheadings helpful or unhelpful?  Did you find the information about 4Pi in the introduction helpful or unhelpful? |

1. **The 4Pi Questionnaire.**

## 4Pi Involvement Experience Questionnaire

#### **What is the questionnaire about?**

Service user and carer involvement is important for improving research and healthcare services. Looking at how involvement is carried out helps to improve the quality and impact of these activities, and the experiences of everyone involved. We define ‘involvement’ as the active participation of service users, carers and stakeholders in shaping, improving or evaluating services. It ensures their voices are heard in decision-making, policy development and service delivery.

The South London and Maudsley (SLaM) NHS Foundation Trust Involvement Team partnered with researchers at [King’s Improvement Science](https://kingsimprovementscience.org/) to develop this questionnaire exploring the experiences of service users and carers taking part in involvement. The questionnaire is based on the [4Pi National Involvement Standards](https://www.nsun.org.uk/projects/4pi-involvement-standards/) which encourage people to think of involvement in terms of: principles, purpose, presence, process and impact. They were developed by the [National Survivor User Network](https://www.nsun.org.uk/) with input from survivors and service users.

**How to complete the questionnaire**

The questionnaire is divided into two sections. It will take approximately 15 to 30 minutes to complete. Section A is about your experience of involvement. In this section you are asked to think about one involvement activity. We appreciate that it may be difficult to choose just one activity if you have been involved in multiple activities. If you would like to provide further information on your experiences, you may of course complete more than one questionnaire on a variety of activities you have been involved in. Section B is about you. We want everyone taking part in involvement to be treated fairly and equally. Asking details about you will help us to see if we are doing this and help to improve how involvement is done.

#### **Why have I been asked to complete the questionnaire?**

You have been asked to complete the questionnaire because you recently took part in involvement activity at the trust [PLEASE ADAPT AS APPROPRIATE]. For example, being involved in reviewing documents or a committee.

#### **Do I have to complete the questionnaire?**

No. It is up to you whether you complete the questions or not. If you choose not to complete these questions the care you receive will not be affected. If you do complete them, the care you receive will not be affected by the answers you provide. All questions are optional. If you do not wish to answer a question, please select ‘prefer not to answer’. You may leave free text boxes blank if you want to, although they do allow you the opportunity to give more detail if you wish. By completing the questionnaire, you are agreeing for the trust to collect, store and use the information that you provide.

#### **What happens to my information?**

#### All information that you share with us will be downloaded and kept securely at [TRUST/ORGANISATION]. [PLEASE INCLUDE HERE ANY APPROPRIATE STATEMENT REFERRING TO DATA MANAGEMENT AND/OR REFERENCE WITH UK GDPR REGULATIONS].

####

#### **Is the questionnaire anonymous?**

Yes, the questionnaire is anonymous. Please do not include any personal information, such as your name, which might identify you or a family member.

#### **Who will see my answers?**

Your answers are confidential and will initially be seen by the patient experience team at the Trust [PLEASE ADAPT AS APPROPRIATE]. Anonymised reports will also be sent to key committees in the Trust that look at the quality and impact of involvement, allowing changes to be made for future involvement activities based on your collective experiences.

#### **Who can I talk to about this questionnaire?**

If you have any questions or if you would like help completing this questionnaire, you can talk to a member of the involvement team at the trust via [PLEASE INSERT EMAIL ADDRESS].

**Thank you for your time.**

### Experience of involvement

### **This section is about a single involvement activity that you have had at the Trust over the last 3 to 6 months. If you have done more than one activity during this time, please choose just one of these. Both positive and negative experiences are welcome.**

### **1.** What single involvement activity at the Trust, over the last 3 to 6 months, would you like to tell us about? (Please only select one option.)

Document review

Advisory group

Committee

Training

Workshop

Other *(please describe)*:

|  |
| --- |

Prefer not to answer

### **Thinking about this involvement activity, please rate your level of agreement with the statements below.** (Please only select one option for each statement. There is a comments box at the end to add any further details or explanations.)

|  | Statement | Strongly disagree | Disagree | Neither agree nor disagree | Agree | Strongly agree | Prefer not to answer | Not applicable |
| --- | --- | --- | --- | --- | --- | --- | --- | --- |
| **2.** | I felt respected and included |  |  |  |  |  |  |  |
| **3.** | My involvement was valued |  |  |  |  |  |  |  |
| **4.** | Service users and carers had the power to influence how things were done. |  |  |  |  |  |  |  |
| *(****Examples****: helping to make decisions about how material is written or presented, or how to run an event.)* | | | | | | | | |
| **5.** | There was a transparent and honest approach to doing things. |  |  |  |  |  |  |  |
| *(****Examples:*** *staff openly discussed challenges and setbacks, staff welcomed and responded to feedback.)* | | | | | | | | |
| **6.** | I understood everyone’s roles, expectations and goals. |  |  |  |  |  |  |  |
| **7.** | I understood what I could and could not influence. |  |  |  |  |  |  |  |
| **8.** | Everyone understood why I was there. |  |  |  |  |  |  |  |
| **9.** | Service users and carers were involved throughout the overall activity. |  |  |  |  |  |  |  |
| **10.** | Service users and carers had different backgrounds and experiences that were relevant to the activity. |  |  |  |  |  |  |  |
| **11.** | I had enough time to prepare. |  |  |  |  |  |  |  |
| **12.** | I had enough information. |  |  |  |  |  |  |  |
| **13.** | I had information that was easy to read and access. |  |  |  |  |  |  |  |
| **14.** | I had enough practical support. |  |  |  |  |  |  |  |
| (**Examples:** printing and posting information, technology support, scheduling meetings at a convenient time, support with travel arrangements, training.) | | | | | | | | |
| **15.** | I had enough support to manage how I felt. |  |  |  |  |  |  |  |
| (**Examples:** help with preparing for meetings, interview panels, help with how to share my story, being able to leave and come back to a meeting if needed.) | | | | | | | | |
| **16.** | I was rewarded appropriately. |  |  |  |  |  |  |  |
| (**Examples:** financial incentive, acknowledgment/ recognition in dissemination outputs i.e., published manuscripts; serving on steering groups/committees) | | | | | | | | |
| **17.** | I received feedback on the results of the activity. |  |  |  |  |  |  |  |
| (**Examples:** through a report, meeting or email.) | | | | | | | | |
| **18.** | I had a positive experience. |  |  |  |  |  |  |  |
| **19.** | My involvement made a difference to the activity. |  |  |  |  |  |  |  |
| **20.** | My involvement makes a difference beyond the activity itself. |  |  |  |  |  |  |  |
| (**Examples:** improving the wellbeing of service users and carers who are involved, helping people to see the value of involvement, helping to improve the organisation.) | | | | | | | | |

**21.**Please add any additional comments below. You could include examples to explain why you chose your answers.

|  |
| --- |

**22.** Is there anything additional that you would like to add about your experiences of involvement at the Trust over the last 3 to 6 months, that you do not feel was covered in the previous questions? Please provide further details.

|  |
| --- |

### About you

### We want everyone taking part in involvement to be treated fairly and equally. We will use the information in the next set of questions to help us to see if we are doing this and understand the diversity which will help improve how involvement is done. Please remember your responses are anonymous, confidential and optional.

**23.** How would you describe your involvement at the Trust? *(Please select all that apply.)*

Service user

Carer

Previous service user

Previous carer

Other

Prefer not to answer

**24.** What is your age range?

18 – 24

25 – 44

45 – 64

65 – 84

85 or more

Prefer not to answer

**25.** What is your gender?

Non-binary/ genderqueer/ agender/ gender fluid

Woman

Man

Intersex

Other *(please describe)*:

|  |
| --- |

Prefer not to answer

**26.** Has your gender identity changed from birth?

Yes

No

Prefer not to answer

**27.** How would you describe your sexual orientation?

Asexual

Bisexual

Gay, Lesbian or Homosexual

Heterosexual (some people refer to this as ‘Straight’)

Pansexual

Other *(please describe)*:

|  |
| --- |

Prefer not to answer

**28.** How would you describe your ethnic origin?

Asian or Asian British – Bangladeshi

Asian or Asian British – Indian

Asian or Asian British – Pakistani

Asian or Asian British – any other Asian background *(please describe)*:

|  |
| --- |

Black or Black British – African

Black or Black British – Caribbean

Black or Black British – any other Black background *(please describe)*:

|  |
| --- |

Mixed – White and Asian

Mixed – White and Black African

Mixed – White and Black Caribbean

Mixed – any other Mixed background *(please describe)*:

|  |
| --- |

Other ethnic group – Chinese

Other ethnic group – Latin-American

Other ethnic group – any other ethnic group *(please describe)*:

|  |
| --- |

White – White British

White – White Irish

White – Gypsy or Irish Traveller

White – any other White background *(please describe)*:

|  |
| --- |

Not known

Prefer not to answer

**29.** What is your religion or belief?

Atheism

Buddhism

Christianity (including Church of England, Catholic, Protestant

and all other Christian denominations)

Hinduism

Islam

Judaism

Sikhism

No religion

Other *(please describe)*:

|  |
| --- |

Prefer not to answer

**30.** Do you consider yourself to have a disability?

Yes

No

Prefer not to answer

**31.** If you answered yes to question 30, please select the type of disability.

*(Please select all that apply.)*

Hearing difficulty

Learning disability/difficulty

(dyslexia, dyspraxia, dyscalculia, sensory processing disorder and others)

Mental health condition

Physical health condition

Neurodiversity (attention deficit hyperactivity disorder, autism and others)

Vision impairment

Other *(please describe)*:

|  |
| --- |

Prefer not to answer

**Thank you for completing this questionnaire.**

1. **GRIPP2 Short Form**. This table report how PPI was involved in each phase of the study

| **Section and topic** | **Item** | **Reported on page No** |
| --- | --- | --- |
| 1. **Aim** | **Report the aim of PPI in the study**  The aim of having PPI in the study was to bring an inner perspective into the development of a tool that will be used to measure service users and carers’ experience in involvement activities. | 8 |
| 1. **Methods** | **Provide a clear description of the**  **methods used for PPI in the study**  PPI representatives were involved across different phases of the study. They were involved in: a) determining face validity of the questionnaire; b) testing accessibility and usability of the questionnaire; c) reviewing the Guide accompanying the questionnaire. | 13 |
| 1. **Study Results** | **Outcomes – report the results of**  **PPI in the study, including both**  **positive and negative outcomes**  PPI contributed to improving clarity of questionnaire statements, reducing their number (face validity). They provided recommendations that were used to produce the final version of the questionnaire (Accessibility and usability phase), and revised the Guide accompanying the questionnaire with feedback on font, format. | 16, 21, 19-21 (Table 3) |
| 1. **Discussion and conclusions** | **Outcomes – comment on the extent to which PPI influenced the study overall. Describe positive and negative effects**  Working along PPI representatives greatly contributed to the development of an instrument that is relevant, and that would produce impact such as rigorous measurement of PPI activities. | 22-23 |
| 1. **Reflections/critical perspectives** | **Comment critically on the study, reflecting on the things that went well and those that did not, so others can learn from this experience**  Participation of PPI representatives was carefully planned to ensure that everyone could contribute, feel valued, and be acknowledged for their work. However, due to limited capacity, PPI representatives were not able to be deeply involved in the analysis of the cognitive interviews, as the research team did not have sufficient resources to provide the preparatory training required for meaningful involvement. | 26 |

1. A Questionnaire Guide to accompany the 4Pi Questionnaire.

**4**

**Pi Involvement**

**Experience**

**Questionnaire**

**Guide**


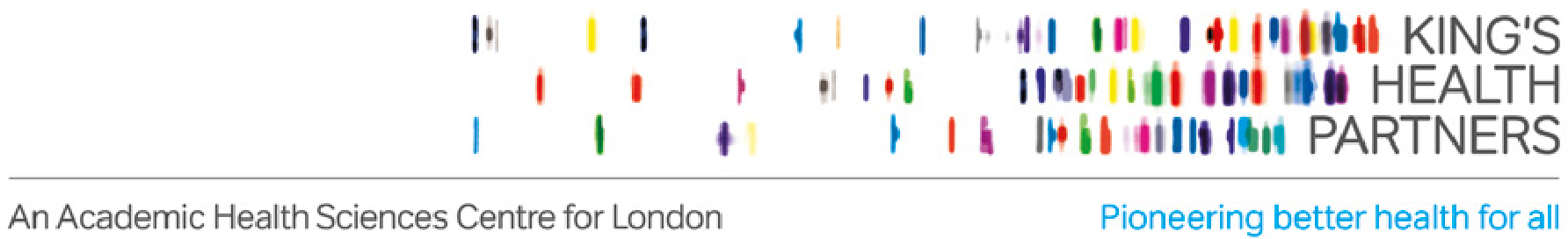


A guide to accompany the 4Pi Involvement Experience

Questionnaire 2025


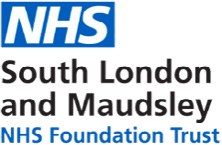

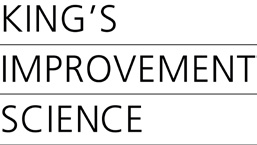


**Foreword**

The 4Pi Involvement Experience Questionnaire and this accompanying guide

were developed by King’s Improvement Science (KIS) in collaboration with the

Improvement Service, Service User and Carer Involvement Team at South London

and Maudsley (SLaM) NHS Foundation Trust. The 4Pi National Involvement

Standards encourage people to think of involvement in terms of:

**principles,**

**purpose, presence, process and impact**

. They were developed by the National

Survivor User Network with input from survivors and service users.

The KIS project team included Kathryn Watson, Manuela Russo, Erin Letbe-

Holder, Hema Chaplin, Fiona Hackett and Bernadette Khoshaba who worked in

collaboration with the Service User and Carer Involvement Lead at SLaM, Richard

Morton.

Four Patient and Public Involvement (PPI) representatives supported the

development of the questionnaire and this guide: Araya Gautam, Charlie Costa,

Chris Pavlakis and Rashmi Kumar.

We would like to thank all the staff, patients and public members that took part in

and contributed to this project. Without their valuable insights and perspectives,

this project would not have been possible.

The study received approval from the Health Research Authority and from the

London Bridge Research Ethics Committee (REC reference 24/PR/0662).

Further information about the 4Pi Questionnaire and this guide can be found on

the King’s Improvement Science website (

[www.kingsimprovementscience.or](https://kingsimprovementscience.org/)

[g](https://kingsimprovementscience.org/)

)

,

along with contact details for the researchers involved in this work. Additionally,

details regarding the procedures and methodology used in the questionnaire

development can be found in Watson et al 2025 (in preparation).

# Contents

**Introduction** 4

Background 4

About this guidance 4

Who this guidance is for 4

Part one: **Developing the 4Pi Questionnaire** 5

Defining and measuring patient and public user involvement activity 5

4Pi Framework 5

Overview of the 4Pi Questionnaire development 6

4Pi Questionnaire structure and domains 8

Part two: **Guidance and recommendations for administering the 4Pi** 10

Questionnaire 10

Guidance and recommendations 10

Questionnaire administration 10

Accessibility 11

**References** 13

# Introduction

## Background

Although the importance of community engagement in research has been previously established, there are few evidence-based approaches for measuring the level of community engagement in research projects. Evaluation of patient and public involvement (PPI) is important to ensure that such involvement activities are conducted with a good degree of quality and in a meaningful way. How best to evaluate PPI remains challenging, given the complex landscape of diverse terminology and multiple methods. This highlights a need for more robust and standardised methods to evaluate PPI, which also involve patients and public members as part of their design process. To meet this need, a self report questionnaire has been developed, based on the 4Pi National Involvement Standards, to assess service users’ (SU’s) and carers’ experiences of PPI.

## About this guidance

This guide has been produced to accompany the 4Pi Involvement Experience Questionnaire, hereafter referred to as the ‘questionnaire’. It contains a summary of the questionnaire’s aim and rationale, questionnaire development process, description of the final questionnaire, and guidance and recommendations for its use.

The questionnaire is a self-report questionnaire that was developed based on the

4Pi National Involvement Standards, commonly referred as the ‘4Pi Framework’ (*Faulkner, 2014; National Survivor User Network. 4Pi Involvement Standards*). The questionnaire aims to capture service users’ (SU’s) and carers’ views on their experiences of undertaking patient and public involvement (PPI) activities. As such, the questionnaire can be used as part of an evaluation of PPI, that is systematic, and applicable in a wide variety of contexts, which is grounded in the experiences of SUs and carers themselves.

## Who this guidance is for

The questionnaire’s intended purpose is to be used by service providers and other stakeholders that carry out patient and public involvement (PPI) activities, who would like to assess the experiences of service users and carers in conducting those activities. This guide provides helpful information on implementing the questionnaire, specifically on administering it and improving its accessibility.

This guidance is divided into two parts. The first part describes the development of the 4Pi Questionnaire, and the second part covers how to use and implement it in your service, institution or trust.

# Part one: Developing the 4Pi Questionnaire

## Defining and measuring patient and public user involvement activity

Patient and public involvement in research (PPI) refers to an activity done ‘with’ or ‘by’ SUs and carers rather than ‘about’, ‘on’ or ‘for’ them (INVOLVE, 2009). PPI in care planning and service improvement is a well-established requirement by the National Health Service (NHS), institutions and funding bodies (NSUN, 2015). Over the past three decades several legislations and policies have been produced to promote, support and strengthen PPI within NHS services, local communities and research (Involvement, 2019).

Robust evaluation is an important means to facilitate bringing out improvements to PPI, and ensuring that best practices are shared, alongside lessons learnt. However, conducting quality and impactful PPI is challenging (Machin et al., 2023). Some guidelines and measures have been produced to facilitate the evaluation of PPI in different contexts (Boote et al., 2006; Gibbons et al., 2014; Morrow et al., 2010; Wright et al., 2010). However, these are not universally applicable and tend to be poor in quality (Gibbons et al., 2014; Goodman et al., 2017).

## 4Pi Framework

In 2013, the 4Pi Framework was developed to provide national standards for good practice, and to monitor and evaluate involvement of SUs and carers in planning, delivery and evaluation of services (Faulkner, 2014).

The 4Pi Framework, developed in partnership with SUs and carers, as part of the

National Involvement Partnership (NIP) project from the National Survivor User Network (NSUN), has universal relevance and covers five domains: Principles, Purpose, Presence, Process and Impact. They are briefly described below:

l **Principles:** Meaningful and inclusive involvement requires a commitment to shared principles and values, such as respect, inclusivity, equality and fairness. This means recognising the contribution of SUs and carers as equally important to those of professionals, while also embracing cultural diversity and promoting race equality.

l **Purpose:** Involvement should have a well-defined purpose that is clearly shared and communicated to all participants and the broader organisation. The core purpose of any involvement activity is to enhance services and improve the experiences of SUs and carers.

l **Presence:** Diversity of SUs and carers is crucial and should be present at all levels and stages of an organisation and within projects, including at decision making levels. SUs and carers should be provided with the opportunity to be involved separately as they might have different priorities from other participants/professionals.

l **Process:** Planning is essential for successful involvement. It should cover recruitment and engagement, communications, appropriate support, training and payment.

l **Impact:** Involvement should bring an improvement to people’s lives. Impact should be explored at different levels, including ethos and culture, policy and planning, delivery of the project, outcomes, diversity and equality opportunities, and experience of services.

Given its universal relevance, clearly defined and systematic approach and grounding in SUs and carers experiences, the 4Pi Framework lends itself well to guide the development of a new measure to evaluate the experience of PPI. Such a questionnaire would serve as a robust, relevant and meaningful tool to facilitate the evaluation of the experience of SUs and carers on involvement activities across many contexts.

More detailed information about the 4Pi Framework and case studies can be found by following these weblinks:

[www.nsun.org.uk/projects/4pi-involvement-standards/](https://www.nsun.org.uk/projects/4pi-involvement-standards/) [www.nsun.org.uk/projects/4pi-involvement-standards/4pi-in-practice/](https://www.nsun.org.uk/projects/4pi-involvement-standards/4pi-in-practice/)

## Overview of the 4Pi questionnaire development

The core project team responsible for questionnaire development consisted of two mixed-methods researchers and two PPI coordinators working in collaboration with a PPI lead at a local mental health NHS foundation trust in south London. Questionnaire development was guided by a practical or ‘what will work best’ approach and was focussed on producing a self-report questionnaire that was accessible, appropriate, meaningful and feasible for application to a wide variety of contexts.

Literature review and expert consultation were utilised for item generation. Following this, iterative cycles of questionnaire review and modification were carried out (Table 1). Input was obtained from both experts (individuals with expertise in applying the 4Pi framework to PPI) and those from the target population (PPI members or SUs and carers).

SUs and carers were involved both as participants of a focus group (to evaluate questionnaire functioning and to improve its comprehensibility) (Crowley et al., 2020; Farmer et al., 2022), but also as part of PPI activities within the project (i.e., group consultations at the beginning and end of questionnaire development, review of this guide, and monthly input on study progress via an internal PPI group).

Questionnaire review included use of a Questionnaire Appraisal System (QAS) (Schaad et al., 2020), which is a checklist designed to aid finding and fixing common questionnaire problems.

The main areas of focus of the group-based cognitive interviews were relevance and representativeness of items to the 4Pi domains, item functioning (i.e. how easy items were to understand and meaningfully answer), questionnaire flow, item reduction, formatting and accessibility. Consensus on questionnaire modifications was reached through discussion among the research team.

The main challenge of questionnaire development was achieving the right balance between comprehensiveness (i.e. satisfactory representation of the 4Pi framework and expectations of PPI members), and respondent fatigue (i.e. the amount of effort required by respondents to complete the questionnaire).

Respondent fatigue mostly related to item complexity and questionnaire length. As such, the final questionnaire does not explicitly group items into 4Pi domains, some initial items were removed, and some others are double-barrelled. Common reasons for item removal were: potential overlap with other items, respondents unlikely to have the knowledge to answer, items more suited to other methods of data collection, low priority weighed against respondent fatigue, or irrelevant to the domain.

**Table 1.** Description of questionnaire development steps

| **Step** | **Name of development step** | **Description** |
| --- | --- | --- |
| 1 | First draft of questionnaire | Domain identification and item generation informed by literature review and expertise within the project team. |
| 2 | Expert input | Consultation with a subject-matter expert (a survivor researcher and co-author of the 4Pi-Framework). |
| 3 | PPI: Face validity | Online focus group consultation with four PPI members, focussed on assessing face validity (i.e. the degree to which the questionnaire is measuring what it is intended to measure). |
| 4 | Questionnaire Appraisal | Evaluation using an adapted version of the |
|  | System (QAS) review | ‘modified QAS”’(Schaad 2020). |
| 5 | Expert review | Consultation with two subject-matter experts (PPI leads at collaborating NHS trust), providing feedback via email and online interview. |
| 6 | Focus group-based | Online focus group-based CI with five PPI |
|  | cognitive interview (CI) | members. |
| 7 | PPI: Accessibility | Group consultation with 11 PPI members, providing feedback via an online form, with a focus on accessibility. |

The final round of questionnaire development or ‘accessibility review’ suggested that the questionnaire was overall easy to understand and complete. However, respondent fatigue may still be an issue for some. The main suggestion offered was to simplify some items, and the main challenge raised was focusing on one involvement activity to fill in the questionnaire.

To increase the validity and reliability of the questionnaire, additional development steps could be carried out. This could include conducting a pilot test with a diverse cross-section of the target population (Ruel et al., 2016), as well as psychometric validation (Boateng et al., 2018).

The 4Pi Questionnaire will provide a snapshot of overall SU and carer involvement experience. Conducting follow-up evaluations (for example, via subsequent questionnaires, interviews or focus groups) could provide more in-depth information about these experiences. In addition, areas where further investigation may be useful include exploring impact at different stages (short, medium and long-term), and exploring whether impacts were expected or unexpected.

More detailed information about payment terms and processes could be important to investigate further as well. Areas not covered explicitly by the questionnaire could also be considered in follow-up studies. For example, to explore SU’s and carers’ experience of recruitment processes, or to capture additional respondent demographics that may influence involvement experience, such as whether respondents had parental or other caring responsibilities.

To provide a wider evaluation of SU and carer involvement experience, additional information could be collected via alternative methods, alongside using the questionnaire. For example, exploring staff experience, including involvement coordinators, managers and researchers where applicable, could complement information gathered from the SU and carer perspective to evaluate that the PPI is carried out at all levels within the organisation, in all projects and at all stages within projects.

## 4Pi Questionnaire structure and domains

The 4Pi Questionnaire consists of an introduction and two sections containing 31 items in total. Completion is estimated to take approximately 20-25 minutes. All items are required to be completed; however, the response ‘prefer not to answer’ is an option for all items. The introduction contains background information to help respondents decide whether they wish to complete the questionnaire.

Section A, ‘Your experience of involvement’ (22 items), is about the involvement experience and Section B, ‘About you’ (9 items), is to collect demographic information.

Section A is designed to collect information about experiences related to a single recent involvement activity. Respondents are asked to specify the type of activity in Item 1. Following this, they are asked to rate their level of agreement to 19 items covering the 4Pi domains (i.e., Principles, Purpose, Presence, Process and

Impact) on a 5-point Likert scale from ‘strongly disagree’ (1) to ‘strongly agree’ (5).

There are also options of ‘prefer not to answer’ and ‘not applicable’. An example of statement is reported below in **Figure 1**.

**Figure 1** Example of statement with choice of responses

|  | | **Statement** | | |  |  |
| --- | --- | --- | --- | --- | --- | --- |
| Strongly | Disagree | Neither | Agree | Strongly | Prefer | Not |
| disagree |  | agree |  | agree | not to | applicable |
|  |  | nor disagree |  |  | answer |  |
|  | | **I felt respected and included** | | |  |  |

Types of involvement activities can vary considerably, ranging from one-off to long-term, individual to group, consultation to co-production, for example. Respondents may also vary in the number of activities they choose to get involved in, as well as in the extent of their involvement.

The decision to focus on a single involvement activity, rather than to ask respondents to generalise their experiences across multiple activities, was made for two reasons. Firstly, to enable experiences to be stratified by activity type, and secondly, to ease questionnaire completion and improve the meaningfulness of responses. However, some individuals, particularly those involved in many involvement activities, may still find this approach challenging.

**Table 2** shows how items in Section A map to their 4Pi domain. The end of Section A contains two free text items to enable respondents to provide further information or explanations.

**Table 2**. 4Pi Questionnaire items mapped to 4Pi domains

| **Item numbers** | **4Pi domain** |
| --- | --- |
| 2–5 | Principles |
| 6–8 | Purpose |
| 9, 10 | Presence |
| 11–17 | Process |
| 18–20 | Impact |

Items in Section B cover SU and carer status, age, gender, sexual orientation, ethnicity, religion and disability. Respondent demographics were included, as stratifying experiences by these characteristics could help identify whether involvement practices are inclusive or whether there is any evidence of marginalisation.

# Part two: Guidance and recommendations for administering the 4Pi Questionnaire

## Guidance and recommendations

This part contains guidance for questionnaire administration which may be helpful to consider when using the questionnaire to evaluate PPI. These suggestions are based on research team discussions, as well as feedback from SUs and carers, and PPI leads or subject matter experts, as part of the questionnaire development process. It also includes potential recommendations for making the questionnaire accessible in paper or online versions.

## Questionnaire administration

The following suggestions relate to planning the evaluation, questionnaire accessibility, and follow-up after an evaluation has taken place.

1. **Review and adapt:** The questionnaire is designed to be applicable to a wide range of contexts. However, it is recommended that individuals and organisations wishing to use the questionnaire review the content and adapt it to individual needs and circumstances, where necessary. For example, in the introductory part of the questionnaire (pages 1-2), a reference to the institutional policies or the name of the organisation, relevant teams within the organisation and contact details will need to be adapted. Additionally, a relevant involvement activity can be added to the list in Question 1. However, modifying any of the statements in Section A and B is discouraged, as these were thoroughly defined during an intensive and iterative development phase.
2. **Resource and embed:** Embed evaluation into the research process and ensure adequate resources are available to analyse and action any feedback collected. Actioning any feedback in a timely manner will improve its relevance and impact.
3. **Multiple responses:** Enabling respondents to feedback on more than one involvement activity is important for some. This could also provide a more holistic overview of SUs and carers’ experiences. To facilitate this, we recommend enabling respondents to submit more than one questionnaire.
4. **Regular evaluation:** Using the questionnaire as part of a regular evaluation and improvement cycle will increase its effectiveness. The timings of evaluation cycles will depend on various factors, including available resources, questionnaire design and respondent characteristics.
5. **Respondent fatigue:** As the questionnaire would approximately take 20 minutes to be filled in, it may not be appropriate for frequent administration due to respondent fatigue (which will more greatly affect people with disabilities, especially those with multiple conditions). Please bear this in mind when planning the frequency of questionnaire administration.
6. **Respondent selection:** Clearly define and agree the questionnaire eligibility criteria. This will ensure that ‘certain kinds’ of respondents are not cherry-picked, which could bias any results obtained.
7. **Support:** Offer tailored support for respondents to complete the questionnaire such as completion over the phone or via one-to-one conversation if the respondent prefers it. At the same time, please to be aware of some potential issues such as social desirability bias of the respondent and/or lack of anonymity.
8. **Feedback loop:** Ensure respondents are informed of the results of evaluation process and the actions taken.

## Accessibility

Accessibility of the questionnaire is essential to ensure it meets needs of all potential respondents including challenges such as vision, hearing and physical/ dexterity difficulties. It will be the responsibility of the involvement team or trust to adhere to their licensing agreements and internal policies, while also complying with legal requirements for data protection and intellectual property rights.

A list of recommendations to improve accessibility is provided below:

l The questionnaire should be available in both digital and paper versions to meet different needs and preferences.

l A digital version of the questionnaire could be developed and distributed using different tools. For example, via email by sending the questionnaire as a modifiable word processing document (e.g., Microsoft Word, Apache OpenOffice Writer), or via a link to an advanced survey platform (e.g., Qualtrics, REDCap, SurveyMonkey Enterprise) or to a survey and form builder (e.g., Google Forms, Microsoft Forms).

l To improve readability both digital and paper versions should avoid breaking-up sentences and/or statements across two pages.

l Response options should be offered in a drop-down menu (digital version) or in a list (paper version). Response options in a grid should be avoided as this can be challenging to read through.

l Response options for demographic details should reflect the trust’s routinely collected demographical information.

l A dark colour font on a white background in at least 12-point (Arial) font should be used.

l Use of italics or bold should be used only for individual words not whole sentences or statements as it makes them harder to read.

l To promote digital accessibility, functions like a progress bar and ‘save-and return’ should be offered for easy navigation and completion.

l Consider producing an Easy Read version of the questionnaire to further increase accessibility.

# References

Boateng, G. O., Neilands, T. B., Frongillo, E. A., Melgar-Quinonez, H. R., & Young, S. L. (2018). Best Practices for Developing and Validating Scales for Health, Social, and Behavioral Research: A Primer. Front Public Health, 6, 149. [https://doi.org/10.3389/fpubh.2018.00149](https://www.frontiersin.org/journals/public-health/articles/10.3389/fpubh.2018.00149/full)

Crowley, T., Van der Merwe, A., & Skinner, D. (2020). Development of a cultural and contextual appropriate HIV self-management instrument using interpretive phenomenology and focus group cognitive interviews. International Journal of Africa Nursing Sciences, 12, 100207. [https://doi.org/https://doi.org/10.1016/j.ijans.2020.100207](https://doi.org/https:/doi.org/10.1016/j.ijans.2020.100207)

Farmer, N., Powell-Wiley, T. M., Middleton, K. R., Brooks, A. T., Mitchell, V.,

Troncoso, M., Ceasar, J., Claudel, S. E., Andrews, M. R., Kazmi, N., Johnson, A., & Wallen, G. R. (2022). Use of a focus group-based cognitive interview methodology to validate a cooking behavior survey among African-American adults [Original Research]. Frontiers in Nutrition, 9. [https://doi.org/10.3389/fnut.2022.1000258](https://www.frontiersin.org/journals/nutrition/articles/10.3389/fnut.2022.1000258/full)

Faulkner, A. Y., Sarah; Kalathil, Jayasree; Crepaz-Keay, David; Singer, Fran; James, Naomi; Griffiths, Raza; Perry, Emma; Forde, Denise; Kallevik, Joyce. . (2014).

4Pi report: involvement for influence full version. [https://www.nsun.org.uk/wpcontent/uploads/2021/05/4PiNationalInvolvementStandardsFullReport20152. pdf](https://www.nsun.org.uk/wp-content/uploads/2021/05/4PiNationalInvolvementStandardsFullReport20152.pdf)

Gibbons, C. J., Bee, P. E., Walker, L., Price, O., & Lovell, K. (2014). Service user- and carer-reported measures of involvement in mental health care planning: methodological quality and acceptability to users. Front Psychiatry, 5, 178. [https://doi.org/10.3389/fpsyt.2014.00178](https://www.frontiersin.org/journals/psychiatry/articles/10.3389/fpsyt.2014.00178/full)

Goodman, M. S., Sanders Thompson, V. L., Johnson, C. A., Gennarelli, R., Drake, B. F., Bajwa, P., Witherspoon, M., & Bowen, D. (2017). EVALUATING COMMUNITY

ENGAGEMENT IN RESEARCH: QUANTITATIVE MEASURE DEVELOPMENT. J

Community Psychol, 45(1), 17-32. [https://doi.org/10.1002/jcop.21828](https://onlinelibrary.wiley.com/doi/10.1002/jcop.21828)

INVOLVE. (2009). The impact of public involvement on research. A discussion paper from the INVOLVE Evidence, Knowledge and Learning working group.

Involvement, U. S. f. P. (2019). UK Standards for Public Involvement in Research. Retrieved 26.02.2025 from [https://sites.google.com/nihr.ac.uk/pi-standards/ standards?authuser=0](https://sites.google.com/nihr.ac.uk/pi-standards/standards?authuser=0)

Machin, K., Shah, P., Nicholls, V., Jeynes, T., Trevillion, K., & Vera San Juan, N. (2023). Co-producing rapid research: Strengths and challenges from a lived experience perspective. Frontiers in Sociology, 8, 996585.

National Survivor User Network. 4Pi Involvement Standards. Retrieved 26 November 2024 from [https://www.nsun.org.uk/projects/4pi-involvementstandards/](https://www.nsun.org.uk/projects/4pi-involvement-standards/)

NSUN, N. S. U. N. (2015). Service User Involvement in Health and Social Care Policy and Legislation (4Pi National Involvement Standards., Issue.

Ruel, E., Wagner, W., & Gillespie, B. (2016). The practice of survey research. SAGE Publications, Inc. [https://methods.sagepub.com/book/edvol/the-practice-ofsurvey-research/toc](https://methods.sagepub.com/book/edvol/the-practice-of-survey-research/toc)

Schaad, A., Jans, M., & Scott, M. (2020). Improving the Question Appraisal System (QAS): Moving Further Away from Black Magic and Black Boxes. American Association for Public Opinion Research.
